# Surveys/Research Exploring Japanese Phase I Studies in Global Drug Development: Are They Necessary Prior to Joining Global Clinical Trials?

**DOI:** 10.1002/cpdd.1044

**Published:** 2021-11-26

**Authors:** Masaru Hirano, Masanori Yamada, Toshiaki Tanaka, Toshiko Koue, Tomohisa Saito, Mitsuo Higashimori, Hisao Ochiai, Junichi Yamamoto, Saori Yaguchi, Sachiko Mita, Katsutoshi Hara, EFPIA Japan Technical Committee PK/PD Task Force

**Affiliations:** ^1^ Translational Medicine Japan, Novartis Institutes for Biomedical Research Novartis Pharma K.K. Tokyo Japan; ^2^ Clinical Pharmacology Asian Hub GlaxoSmithKline K.K. Tokyo Japan; ^3^ Clinical Sciences, Research & Development Japan Bayer Yakuhin Ltd. Tokyo Japan; ^4^ Clinical Pharmacology Department Chugai Pharmaceutical Co., Ltd. Tokyo Japan; ^5^ Science & Data Analytics, R&D AstraZeneca K.K. Osaka Japan; ^6^ Non‐Clinical, Chemistry, Manufacturing and Control Development & Translational Medicine, Research & Development Institute Merck Biopharma Co., Ltd. Tokyo Japan; ^7^ Clinical Pharmacology, Strategy & Planning for East Asia Region UCB Japan Co., Ltd. Tokyo Japan; ^8^ Current address: Clinical Pharmacology Research & Development Biogen Japan Ltd. Tokyo Japan; ^9^ Clinical, Medical, Regulatory Development Division Novo Nordisk Pharm Ltd. Tokyo Japan; ^10^ Clinical Pharmacology Department, Quantitative Sciences Division, R&D Janssen Pharmaceutical K.K. Tokyo Japan

**Keywords:** ethnic sensitivity/difference, dosage regimen, drug dose, Japanese, phase I study, regulatory science

The pharmacokinetic (PK) and the pharmacodynamic (PD) properties of a drug and correlations of PK and PD with clinical efficacy and safety are important to assess the sensitivity of a drug to potential differences between ethnic groups. Interethnic differences in PK have been observed, often reflecting the well‐characterized differences in body weight across populations.^1^, ^2^, ^3^ In some cases, population differences in pharmacokinetics are associated with interethnic differences in the frequencies of genetic variants of functional consequence for relevant drug metabolizing enzymes and drug transporters (eg, cytochrome P450 [CYP] 2C9, CYP2C19, CYP2D6, organic anion transporting peptide 1B1 and ATP‐binding cassette G2) which in turn may be of clinical consequence.^4^

In some East Asian countries, an assessment of ethnic sensitivity between Asians and non‐Asians is to be included in new drug applications, since there is a concern that there may be interethnic differences in the safety, efficacy, and optimal dosage regimen of the new drug. The International Council for Harmonization (ICH) E5 guideline, “Ethnic Factors in the Acceptability of Foreign Clinical Data” states that drug development harmonization is based on the premise of the potential to waive a redundant study in a new region when foreign clinical data can be accepted as full or partial support for approval in that new region. With regard to the acceptance of foreign clinical data, a critical question to be addressed is whether the foreign clinical data package (eg, results in Western subjects) can be extrapolated to the population of the new region (eg, Asian subjects).

Focusing on Japan, there have been several regulatory guidances related to the participation of Japan in global clinical studies as well as ethnic sensitivity assessment. The notification entitled “Basic Principles on Global Clinical Trials” based on ICH E5 was issued by the Pharmaceuticals and Medical Devices Agency (PMDA) in 2007. This notification recommended that phase I studies should generally be conducted to investigate the safety and PK for drugs in Japanese subjects before Japan joins later‐phase global clinical studies. The 2012 “Reference Cases” update was added to the notification to promote further understanding of the guideline and provided a general overview of conditions for running a Japanese phase I (J‐PhI) study. In addition, the notification “Basic Principles for Conducting Phase I Trials in the Japanese Population Prior to Global Clinical Trials” was issued in 2014. In this notification, further details to be considered when deciding whether to conduct a phase I trial in Japanese subjects were provided, although there were no changes in the principles presented relative to the 2012 update. This notification indicated once again that a J‐PhI study needs to be conducted before Japan can participate in a multiregional clinical trial (MRCT), although, in principle, it might be scientifically acceptable to not require PK and safety data in Japanese participants before Japan joins an MRCT.

For global drug development, the first‐in‐human (FIH) study is generally conducted in the Western region, particularly in multinational pharmaceutical companies, confirming the safety and tolerability of a drug in non‐Asian subjects. A review of phase I studies reported between 2001 and 2009^5^ indicated that ethnic factors do not significantly affect a drug's safety and tolerability profile. Therefore, this initiative questions whether it may be acceptable to not conduct a J‐PhI study (first‐in‐Japanese) prior to participating in global phase II/III studies.

This article presents the integrated results from 2 surveys conducted by the European Federation of Pharmaceutical Industries and Associations (EFPIA) Japan Technical Committee PK/PD Taskforce (EFPIA‐J PK/PD TF) investigating the current status of J‐PhI studies in drug development in Japan over the 2009 to 2019 time frame. The surveys capture the current status of J‐PhI study conduct, J‐Ph1 waiver applications and evaluation of potential for ethic differences across approved drugs.

## Methods

The EFPIA‐J PK/PD TF team includes representatives from AstraZeneca K.K.; Bayer Yakuhin Ltd.; Chugai Pharmaceutical Co. Ltd.; GlaxoSmithKline K.K.; Janssen Pharmaceutical K.K.; Merck Biopharma Co., Ltd.; Novartis Pharma K.K.; Novo Nordisk Pharm Ltd.; and UCB Japan Co., Ltd. (alphabetical order).

The following 2 surveys and research were performed: survey 1: current information on J‐PhI studies in Japan; and survey 2: J‐PhI study waiver applications submitted to the PMDA and their outcomes, and research about information on the ethnic sensitivity of drugs approved in Japan from 2009 to 2019. Survey/research responses were sought from the members in the 9 pharmaceutical companies belonging to the EFPIA‐J PK/PD TF team.

In this initiative, “J‐PhI study” is defined as the first study in healthy subjects of Japanese ancestry for a nononcology indication, with the exception of studies of changes in the route of administration (eg, from intravenous to subcutaneous).

### Survey 1

Survey 1 was a questionnaire on information of current practice in conducting J‐PhI studies prior to patients in Japan participating in a global phase II/III trial:

1‐1: Are phase I studies in Japanese subjects for nononcology or oncology drugs conducted inside or outside Japan?

1‐2: For nononcology drugs, was a J‐PhI study waiver discussed with the PMDA?

### Survey 2

Survey 2 was a questionnaire on J‐PhI study waiver discussions with the PMDA. The following information was obtained: timing of discussion with the PMDA (phase IIa, phase IIb, and/or phase III), drug type (small molecule, monoclonal antibody [mAb], peptide or protein, etc), therapeutic area, whether the indication was a rare disease, whether the mode/mechanism of action (MoA) was novel, route of administration, proposed rationale for the waiver (safety, PK, rare disease, etc), and outcome (“accepted” or “not accepted”) along with the reasons provided by PMDA.

Waivers obtained for the J‐PhI study prior to joining an MRCT were categorized as follows: (1) able to participate in phase IIa/b and/or phase III study directly without any Japanese data or with Japanese data determined via another route(s) of administration, (2) conduct a J‐PhI study in parallel with later phase studies, and (3) include lead‐in/run‐in design for Japanese patients in later‐phase studies.

### Research

The team searched for information on the ethnic sensitivity of drugs approved in Japan over a period of 10 years (2009‐2019) from the 9 companies represented by the EFPIA‐J PK/PD team.

1. Ethnic sensitivity information including PK data were collected for each drug. The following drugs approved between 2009 and 2019 were excluded: (a) ethnic sensitivity not evaluated (eg, no relevant PK data available); (b) oral combination drugs not requiring ethnic sensitivity evaluation, as it was assessed for each molecule previously; (c) drugs approved as active pharmaceutical ingredients before 2009; (d) vaccines and HIV drugs exempted from ethnic sensitivity requirements; and (e) new treatment modalities (eg, regenerative medicine).
2. The assignment of a drug to be ethnically “sensitive” or “not sensitive” was based on the clinical dose approved in the Japanese and non‐Japanese populations. The PMDA review reports and product information for approved drugs in Japan were obtained from the PMDA website (http://www.pmda.go.jp). Information on the ethnic sensitivity assessment and PK parameters was obtained from the product information and the review reports on the PMDA website (http://www.pmda.go.jp/PmdaSearch/iyakuSearch/). The product information for drugs approved in the United States and European Union was obtained from the Food and Drug Administration (https://www.accessdata.fda.gov/scripts/cder/daf/) and European Medicines Agency (https://www.ema.europa.eu/en) websites, respectively, and the information recorded included dose and administration and posology and method of administration.

If the drug dose and dosage regimen approved in Japan were different from those approved in the United States and European Union, the following additional information was obtained:

1. Magnitude of the PK difference between Japanese (or Asian) and non‐Japanese (or non‐Asian) subjects based on the noncompartmental analysis (NCA) results of phase I studies
2. Potential for body weight to explain any PK difference reported
3. The drug‐metabolizing enzymes and drug transporters identified as potentially contributing to disposition
4. Whether a difference in the safety profile was observed between Japanese (or Asian) and non‐Japanese (or non‐Asian) subjects in the phase I studies
5. Whether the difference in PK (or PK/PD) was clinically relevant
6. Dose investigated in Japanese subjects in phase IIb
7. Dose investigated in Japanese subjects in phase III

## Results

### Survey 1

**Figure 1:**
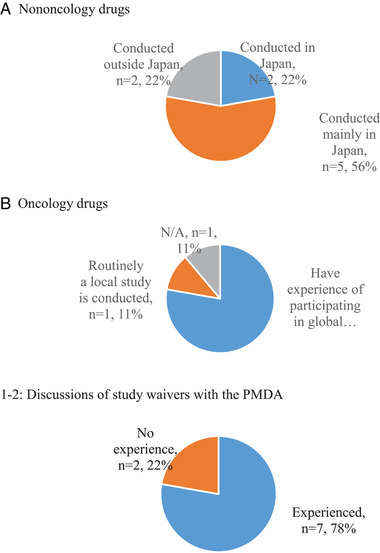
Information about current status of Japanese phase I studies in Japan (N = 9; survey 1). “Conducted mainly in Japan” means a J‐PhI study is usually conducted in Japan, but sometimes Japanese subjects participate in an FIH study conducted outside Japan or a separate J‐PhI study is conducted outside Japan. FIH, first‐in‐human; N/A, not applicable (as 1 company did not develop any oncology drugs); PMDA, Pharmaceuticals and Medical Devices Agency.

As presented in Figure 1, all companies are conducting phase I studies for nononcology drugs in Japanese subjects inside or outside Japan. This aligns with the Japanese regulatory authority requirements and is expected. However, 7 of 9 companies are applying for waivers of the J‐PhI study while others routinely run J‐PhI studies because only a limited number of waivers have been granted to date. Alternatively, for oncology drugs, 7 of the 9 companies surveyed (1 company did not have any oncology assets) have experience in participating in global phase I/FIH trials, which are now becoming common in Japan.

### Survey 2

Waivers of a J‐PhI study were assessed for 22 drugs submitted to the PMDA by the 9 companies included in the survey. The outcomes of the waiver applications and the main reasons provided are shown in Table 1. The reasons provided when the waivers were accepted (n = 13, 59%) were “should not be administered to healthy subjects” (n = 6) and “demonstrated ethnic similarity between Japanese and non‐Japanese for a drug with the same MoA” (n = 4). For these 4 drugs, there were few specific concerns for the Japanese population based on the safety profile and the dose‐proportional PK observed in phase I and/or other clinical studies in non‐Japanese subjects. The other reason for a J‐PhI study waiver was “demonstrated safety in Japanese by another dosing route” (n = 3) indicating previous experience of the drug in Japanese (from oral to intravenous, from oral to intramuscular, and from oral to ocular instillation). On the contrary, the reasons provided for “Not Acceptance” of a Phase I waiver (n = 9, 41%) were that the safety in Japanese could not be estimated in Japanese participants based on the available data. Although an appropriate safety profile and dose‐proportional PK were observed in the phase I or other clinical studies in non‐Japanese participants, and the PK was expected to be similar in Japanese and non‐Japanese populations (eg, mAb, CYP3A‐mediated metabolism), the existing data were considered insufficient to support a phase I waiver (n = 8). The additional reason that a waiver was not accepted was that safety in Japanese subjects after intravenous dosing was to be investigated despite similar PK being observed in Japanese and non‐Japanese following subcutaneous administration.

**Table 1:** Survey of Japanese Phase I Study Waiver Applications Submitted to the PMDA (Survey 2; Total: 22 Cases)

Reason for “Not Accepted”	Cases
Safety concerns in Japanese	9

Reason for “Accepted”	Cases
*Total “accepted”*	*13*
Should not be administered to healthy subjects	6
Demonstrated ethnic similarity for the same MoA drugs	4
Demonstrated safety in Japanese via another dosing route	3

MoA, mode/mechanism of action; PMDA, Pharmaceuticals and Medical Devices Agency.

The rationales proposed for the J‐PhI study waivers included in the survey nominated multiple reasons, including molecule characteristics. These rationales included: (1) safety profile demonstrated in non‐Japanese, (2) dose‐proportional PK demonstrated in non‐Japanese, (3) similar PK (and PD) expected in Japanese and non‐Japanese (eg, for mAb or elimination via nonpolymorphic drug‐metabolizing enzymes such as CYP3A4), (4) PK comparison between Japanese and non‐Japanese planned in MRCTs (eg, population PK) and/or a separate local clinical study proposed in Japanese in parallel with the global phase IIb or III study, (5) no ethnic difference in the safety (and PK) observed for drugs with the same MoA, (6) safety profile and the expectation that the systemic exposure will be within the range of results in Japanese for other routes of administration, (7) local administration (eg, intravitreal injection, ocular instillation), (8) small patient numbers in Japan (eg, rare diseases including oncology indications), and (9) inappropriate to administer to healthy subjects. Table 2 shows the results of the survey of J‐PhI study waiver applications submitted to the PMDA. The acceptance of J‐PhI study waivers did not depend on the therapeutic area of the drugs’ indication. There was a trend for accepted cases to be drugs, with an MoA that was “not novel,” mAbs, or for topical administration and therefore with limited systemic exposure (eg, ocular instillation, intravitreal injection, inhalation). Focusing on the 9 drugs that were mAbs, the 6 cases that were accepted referred to at least 1 reason shown in Table 1, while the 3 rejected cases did not. For small‐molecule drugs, 5 drugs were successfully waived (dosing route: inhalation, ocular instillation, intramuscular, intravenous or subcutaneous injection) due to at least 1 reason in Table 1. For 5 drugs, waivers that were rejected were for 4 oral agents and 1 eyedrop, which could not give a clear rationale for safety in Japanese subjects. It could not be evaluated whether a rare disease indication was associated with waiver success due to the limited number of reports.

**Table 2:** Survey of the Outcome of Japanese Phase I Study Waiver Applications Submitted to the PMDA (Survey 2)

Property	Categories	“Accepted”	“Not Accepted”	Total
Timing of discussion with PMDA	Phase IIa	5	1	6
	Phase IIb	5	7	12
	Phase III	3	1	4
Therapeutic area	Immunology	2	2	4
	Neuroscience	2	2	4
	Autoimmune inflammatory disease	2	0	2
	Ophthalmology	2	1	3
	Infectious disease	2	0	2
	Cardiovascular and metabolism	1	2	3
	Hematology	1	2	3
	Oncology	1	0	1
Rare disease or not	Rare disease	2	2	4
	Not rare disease	11	7	18
Mode/Mechanism of action	New	4	4	8
	Not new	9	5	14
Drug type	Monoclonal antibody	6	3	9
	Small molecule	5	5	10
	Other	2	1	3
Route of administration	Ocular instillation	1	1	2
	Intravitreal injection	1	0	1
	Intra‐articular injection	1	0	1
	Inhalation	2	0	2
	Intramuscular injection	1	0	1
	Oral	0	4	4
	Subcutaneous injection	5	1	6
	Intravenous injection	2	2	4
	Subcutaneous and Intravenous injection	0	1	1

PMDA, Pharmaceuticals and Medical Devices Agency.

### Research

A total of 74 drugs were launched in Japan from 2009 to 2019 by the companies represented in the EFPIA‐J PK/PD TF team, and each drug was evaluated for ethnic sensitivity by the PMDA. For most of the drugs (n = 69, 93.2%), both the sponsor and PMDA acknowledged little or no clinically relevant ethnic sensitivity. This included drugs for which ethnic similarity or difference could not be judged due to the limited data available. Five drugs (6.8%) were reported to have an interethnic difference in PK. Information for these 5 drugs is presented in Table 3: drug PK determinant (metabolizing enzymes and transporters), effect of body weight on PK, safety results, development strategy (ie, local or global phase II/III studies). Of the 5 drugs for which an interethnic difference in PK was reported, there was a different clinical dose for 4 drugs and a different dose regimen for 3 drugs between Japanese and non‐Japanese in phase II and phase III studies. According to the NCA results of the phase I studies, the areas under the plasma concentration–time curve (AUCs) in the Japanese (or Asian) subjects were 1.4‐ to 1.9‐fold higher than the AUCs in the non‐Japanese (or non‐Asian) subjects. Except for ibandronate, the PK differences could not be completely explained by body weight and known differences in drug‐metabolizing enzymes or transporters. Two of the drugs reported significant interethnic differences in safety in the phase I studies (simeprevir and eltombopag). For all 5 drugs, phase IIb and III studies were conducted locally in Japan (except an MRCT for ticagrelor for an old indication, myocardial infarction). Local Japanese phase IIb and III studies were conducted for simeprevir, rivaroxaban (indication: nonvalvular atrial fibrillation), ibandronate, and ticagrelor (indication: acute coronary syndrome) while a combined local phase IIb/phase III study was performed for eltrombopag (indication: idiopathic thrombocytopenic purpura).

**Table 3:** Characteristics of Drugs With Potential Interethnic Difference in Japanese Ancestry on PK: Drugs With PK Differences Between Japanese and Non‐Japanese (Research)

Questions	Simeprevir (Olysio)	Rivaroxaban (Xarelto)	Ticagrelor (Brilinta)	Ibandronate (Bonviva)	Eltrombopag (Revolade)
Dose in package insert (J, non‐J)	J: 100 mg once daily; non‐J: 150 mg once daily	J: 15 mg once daily; non‐J: 20 mg once daily	J and non‐J: 90 mg twice daily (ACS), 60 mg twice daily (OMI)	J: 100 mg once monthly; non‐J: 150 mg once monthly	J: 12.5 mg once daily; non‐J: 50 mg once daily (ITP, initial dose)
PK difference in phase I NCA	1.6‐fold higher in J	1.4‐1.5 fold higher in J	1.4‐fold higher in Asian	1.5‐fold higher in J	1.9‐fold higher in J
BW can explain PK difference	Yes, but partially	Yes, but partially	Yes, but partially	Yes, plausible	Yes, but partially
Drug metaboliz‐ing enzyme/transporter	CYP3A4/5, OATP1B1	CYP3A4/ CYP2J2, ABCB1, ABCG2	CYP3A4/5, ABCB1	Mainly renal excretion	CYP1A2, CYP2C8, UGT1A1, UGT1A3, ABCG2
Significant safety difference in phase I	Yes, higher bilirubin level in J	No	No	No	Yes, platelet count increase in J
Selected dose in phase IIb	J: ≈100 mg once daily; non‐J: ≈150 mg once daily	J: ≈20 mg once daily; non‐J: ≈40 mg once daily	The same as global due to similar PD	The same (J and non‐J: ≈150 mg once monthly)	J: ≈12.5 mg once daily; non‐J: 25 mg once daily^a^
Selected dose in phase III	J: 100 mg once daily; non‐J: 150 mg once daily	J: 15 mg once daily; non‐J: 20 mg once daily	The same as global due to similar PD	J: 100 mg once monthly; non‐J: 100, 150 mg once monthly	

ABC, ATP‐binding cassette; ACS, acute coronary syndrome; BW, body weight; CYP, cytochrome P450; ITP, idiopathic thrombocytopenic purpura; J, Japanese; NCA, noncompartmental analysis; non‐J, non‐Japanese; OAT, organic anion transporter; OMI, old myocardial infarction; PD, pharmacodynamics; PK, pharmacokinetic; UGT, uridine 5′‐diphospho‐glucuronosyltransferase

For all compounds, phase IIb and phase III studies in Japanese were locally conducted.

Information collected for the following 74 drugs (alphabetical order): abiraterone, ambrisentan, apalutamide, artemether/lumefantrine, atezolizumab, avelumab, bedaquiline, belimumab, benralizumab, canakinumab, catridecacog, ceritinib, cetrizumab pegol, cetuxmab, choriogonadotropin alfa, dabrafenib, dapagliflozin, daratumumab, darolutamide, dutasteride, eltrombopag, entrectinib, epoetin beta pegol, esomeprazole, exenatide, fingolimod, fulvestrant, galantamine, glycopyrronium, golimumab, guselkumab, ibandronate, ibrutinib, indacaterol, insulin icodec, lacosamide, levetiracetam, liraglutide, mekinist, mepolizumab, nepafenac, nilotinib, nonacog beta pegol, obinutuzumab, ofatumumab, olaparib, omarizumab, osimertinib, paiperidone, paliperidone palmitate, panobinostat, pasireotide, pazopanib, pertuzumab, ranibizmab, regorafenib, riociguat, rivaroxaban, rivastigmine, romosozumab, ruxolitinib, satralizumab, secukinumab, simeprevir, tapentadol, tedizolid, ticagrelor, trastuzumab‐emtansin, turoctocog alfa, turoctocog alfa pegol, usutekinumab, vandetanib, vemurafenib, vildagliptin.

^a^ Combined phase II/phase III study.

## Discussion

Generally, it is necessary for sponsors to conduct a dedicated phase I study to evaluate the safety and PK of a new drug in a small number of healthy Japanese subjects prior to joining an MRCT. Since the adoption of the ICH E5 guideline in Japan, multinational as well as domestic pharmaceutical companies have accumulated much information on new drug ethnic sensitivity. This knowledge presents an opportunity to consider a new approach to early drug development in Japan, focusing on the phase I studies in healthy Japanese subjects, especially for nononcology indications. For oncology drugs, Japan often joins the FIH trial without first studying any healthy Japanese volunteers. Consequently, oncology drug development in Japan can proceed at the same time as global development. On the other hand, for nononcology other therapeutic areas, a J‐PhI study generally needs to be conducted before a patient can participate in clinical trials. Usually, the FIH study is conducted in non‐Japanese participants, and a separate J‐PhI study is subsequently conducted. This EFPIA‐J PK/PD TF initiative has investigated whether the J‐PhI study in healthy volunteers could be waived if the phase I study data in non‐Japanese healthy subjects is available and obtaining further data in Japanese patients would be more informative than studying healthy volunteers of a second ethnic group.

First, an industry survey of phase I studies conducted in subjects of Japanese ancestry was performed. As expected, for nononcology drugs, J‐PhI studies have been conducted either inside or outside Japan and after, or in parallel with, the global FIH study. In some cases, Japanese subjects are enrolled in the FIH study conducted outside Japan in order for the results to be available prior to initiation of an upcoming MRCT. In contrast, for oncology drugs, most companies can include patients in Japan in global phase I/FIH studies, following a of nonclinical data review by the PMDA prior to study initiation. In addition, it is not ethical to study some nononcology drugs in healthy subjects due to the safety profile, and these drugs should be studied in patients. In these studies, biomarker responses related to the disease can also be investigated. Therefore, for drugs which should not be administered to healthy subjects, a J‐PhI study could be waived, following discussions with the PMDA (Table 1), as it will be possible to determine the safety and PK profile in a small number of Japanese patients in a global phase IIa or phase IIb study.

In most cases, a J‐PhI study waiver is considered after the results of the global FIH study have been obtained. When the safety and PK including dose proportionality following single and/or multiple ascending doses have been determined in non‐Japanese subjects, a primary rationale for the waiver of a J‐PhI study needs to be established without any Japanese safety and PK data being available. To lead this discussion of a waiver with the PMDA, the PK in Japanese subjects should be predicted, using the non‐Japanese data from the FIH study. Furthermore, if available, information on the ethnic similarity of safety/response should be included for other drugs with the same MoA (marketed or under development). This would enable the safety of the new drug in Japanese subjects to be commented on, in relation to the PK predicted.

However, even if the safety profile and dose‐proportional PK are available from phase I or other clinical studies in non‐Japanese subjects and similar PK is expected in Japanese and non‐Japanese subjects (eg, mAb, CYP3A4‐mediated metabolism), this may not be an adequate justification to waive a phase I study from a regulatory perspective. Indeed, although companies have focused phase I study waivers on drugs with little or no ethnic differences from the scientific perspective, some phase I study waivers were rejected due to a lack of assurance of drug safety in Japanese subjects. Therefore, this indicates that the expectation for the J‐Ph1 study is safety confirmation in Japanese, and because of that, the authors think that the contribution of J‐PhI study to determining the ethnic difference in safety profile should be reevaluated (discussed later in the discussion of research). As noted previously in the methods, new modalities such as regenerative medicine are out of scope of this initiative as traditional drugs are discussed. However, waivers of J‐PhI studies have been accepted for some regenerative medicines such as gene and cell therapies based on a discussion of ethnic similarity with the PMDA, although the data discussed are not yet publicly available.

In the case of a change in the route of administration, availability of appropriate safety data in Japanese populations is key. In this survey, 4 such cases were identified; for 3 drugs (oral to intravenous, oral to intramuscular, and oral to ocular injection) J‐PhI study waivers were accepted, but for 1 drug (subcutaneous to intravenous) the waiver was not accepted. Profiling these cases, a study waiver for a new route of administration would be acceptable if the projected systemic exposure is within the range of the exposure observed for the original dosing route and the safety profile observed in Japanese is acceptable. For the 1 rejected case, safety after intravenous administration was confirmed in non‐Japanese subjects but at a lower dose than in the global study planned. The waiver for an intravenous study was not granted because of a lack of non‐Japanese data assuring the safety.

According to the research, in most cases (69/74; 93.2%) there were no clinically meaningful ethnic differences between Japanese and non‐Japanese populations at the dose approved. This was consistent with previous reports.^6^, ^7^, ^8^, ^9^ We also echo the previous reports ^2^, ^3^, ^10^ that PK of mAbs are similar in Japanese and non‐Japanese. In general, mAbs are not widely distributed to organs/tissues, and elimination is not related to the expression level and activity of drug‐metabolizing enzymes and transporters, 2 characteristics associated with low ethnic sensitivity. However, J‐PhI study waivers were not accepted for all mAbs, as presented in Table 2. As described above, it is still considered challenging to predict the safety profile in Japanese even if the PK can be predicted from non‐Japanese data, suggesting that PK alone is not accepted as a surrogate for drug safety. For all drugs, including mAbs, the safety in Japanese subjects should be assessed by considering additional scientific perspectives, including the PD properties of the drug.

In this research of drugs approved in Japan, some compounds that are potentially sensitive to ethnic factors were identified, as shown in Table 3. The PK of these drugs (5/74 drugs; 6.8%) was regarded as ethnically sensitive based on the NCA results of phase I study data. The PK differences reported could not be completely explained by interethnic differences in body weight or by known polymorphisms of the drug‐metabolizing enzymes or transporters identified for these drugs. Considering that there are reports of potential interethnic differences in PK for organic anion transporter P1B1, ATP‐binding cassette G2, and uridine 5′‐diphospho‐glucuronosyltransferase 1A1 substrates between Asians and non‐Asians,^11^, ^12^, ^13^ further investigation is needed to understand the mechanisms responsible. In terms of the clinical doses recommended, the same dose/dosage regimen of ticagrelor was selected by the global and local late‐phase studies in Japan, since similar PD was largely observed at the same dose despite the ≈40% difference in exposure.^14^ A different clinical dose is recommended in Japanese and non‐Japanese populations (European Union, United States) for 4 drugs investigated in local late‐phase studies in Japan. It is not surprising that different studies (local Japan vs global pivotal phase III) investigating different dose/dose regimens would result in different approved doses between Japanese and non‐Japanese.^5^ Looking at the dose/dose regimens selected for phase II studies in Table 3, on the whole, safety appeared to be an important driving factor. Therefore, a PK difference observed in phase I studies would not be the main factor leading to a different clinical dose being selected in Japanese vs non‐Japanese populations. For ticagrelor and ibandronate the doses selected by local phase IIb studies and the global phase IIb study were the same, even though exposures in Japanese subjects were slightly higher than in non‐Japanese subjects. For simeprevir and eltrombopag the interethnic differences in exposure observed in the phase I studies were reflected in different dose regimens being investigated in the phase IIb studies, but again this aligns most with safety signals.

The case of rivaroxaban was complex. The AUC and maximum plasma concentration were 1.4 to 1.5 times higher in the Japanese than non‐Japanese phase I studies. No clear dose‐response relationship was observed in the phase II study, resulting in a weak rationale for setting the dose to be investigated in phase III. The phase III dose in Japan (15 mg) was selected to attain the same exposure as in the global study (20 mg), in anticipation of achieving the same efficacy, assuming no relevant interethnic difference in PD response. In addition, the medical practice in Japan for anticoagulant therapy in the target disease was also assessed to be similar to global medical practice. The final dose selected for Japanese patients was based on the efficacy and safety data from the Japanese phase III study. This example suggests that it would be meaningful to conduct a J‐PhI study if Japanese‐specific safety concerns were anticipated. However, in most cases, it is not easy to exclude potential interethnic differences in safety as a consequence of the small number of subjects studied in Phase I, especially if genetic factors may be important. Phase I studies are typically conducted in 6 to 8 healthy subjects per dose cohort, indicating there is low statistical power to detect low‐frequency safety events.^7^ Furthermore, if notable interethnic differences are not observed, the safety results of the J‐PhI study do not contribute information to dose adjustments in later larger multinational phase II and phase III studies. Accordingly, the EFPIA‐J PK/PD TF team question whether a J‐PhI study is needed for all new molecular entities, specifically when no interethnic difference in PK is expected. In such situations, the safety and PK in Japanese would be more conclusively evaluated in patients in the global phase IIb study. With sentinel dosing, careful safety monitoring (eg, hospitalization if needed) and intensive PK sampling with population PK analysis as appropriate, the safety profile and PK can be evaluated in phase II patient studies. In our opinion, this is also a more optimal approach even if the drug is regarded as potentially ethnically sensitive.

This report is based on the activities of the EFPIA‐J PKPD TF team between 2017 and 2019. A limitation is that information was collected from just 9 pharmaceutical companies. However, the general findings and proposals are consistent with those previously published.^2^, ^3^, ^7^, ^9^, ^10^ The authors hope that this report will lead to further discussion on this topic with other pharmaceutical companies, academia, and regulatory agencies.

## Conclusions

Drug development programs now commonly include Japan in MRCTs. Currently, to accelerate development timelines and new drug application filings, various development strategies are considered rather than the traditional approach of phase I, phase IIa, phase IIb, and then a phase III study. To be included in the global development of nononcology drugs, Japan should be included in early development strategies, either by including a J‐PhI study or based on a rationale for a waiver of a J‐PhI study. In general, Japan could be more often included in global development and participate in MRCTs earlier if the J‐PhI study could be waived. As discussed, additional flexibility and expansion of J‐PhI study waivers for drugs expected to have low ethnic sensitivity should be considered when supported by an adequate scientific rationale.

ICH E5 states “for regions with little experience with registration based on foreign clinical data, the regulatory authorities may still request a bridging study for approval even for compounds insensitive to ethnic factors. As experience with interregional acceptance increases, there will be a better understanding of situations in which bridging studies are needed. It is hoped that with experience, the need for bridging data will lessen.” Over the past 22 years, there has been extensive experience in assessing new drugs and their interethnic PK as well as the ethnic sensitivity of safety and efficacy in Japan. Therefore, we encourage an update to guidance with respect to when phase I studies are truly needed and J‐PhI study waivers are acceptable in Japan.

## Conflicts of Interest

M.H. is a current employee of Novartis Pharma K.K. K.H. and M.Y. are current employees of GlaxoSmithKline K.K. T.T. and T. K. are current employees of Bayer Yakuhin Ltd. T.S. is a current employee of Chugai Pharmaceutical Co., Ltd. M.H. is a current employee of AstraZeneca K.K. H.O. is a current employee of Merck Biopharma Co., Ltd. J.Y. was an employee of UCB Japan Co., Ltd. at the time the study was conducted. S.Y. is a current employee of Novo Nordisk Pharm Ltd. S.M. is a current employee of Janssen Pharmaceutical K.K. Writing of this paper did not require any separate funding, and the authors declared no conflict of interest above their employment with the pharmaceutical companies indicated.

## Data Sharing

The data used in this paper are not accessible.

## Appendix

International Council for Harmonisation of Technical Requirements for Pharmaceuticals for Human Use. ICH E5: International Conference on Harmonisation. Ethnic factors in the acceptability of foreign clinical data E5 (R1). https://www.pmda.go.jp/files/000156836.pdf; 1998 [accessed Nov 2021].

Japanese Ministry of Health Labour and Welfare. Basic principles on global clinical trials. Notification 0928010 (Sep. 28, 2007). https://www.pmda.go.jp/files/000153265.pdf; 2007 [accessed Nov 2021].

Japanese Ministry of Health Labour and Welfare. Basic Principles on Global Clinical Trials (Reference Cases) https://www.pmda.go.jp/files/000152969.pdf; 2012 [accessed Nov 2021].

Japanese Ministry of Health Labour and Welfare. Basic Principles for Conducting Phase I Trials in the Japanese Population Prior to Global Clinical Trials. https://www.pmda.go.jp/files/000157777.pdf; 2014 [accessed Nov 2021].
